# Fasting and rapamycin: diabetes versus benevolent glucose intolerance

**DOI:** 10.1038/s41419-019-1822-8

**Published:** 2019-08-13

**Authors:** Mikhail V. Blagosklonny

**Affiliations:** Cell Stress Biology, Roswell Park Cancer Institute, Elm and Carlton Street, Buffalo, NY 14263 USA

**Keywords:** Diseases, Endocrinology

## Abstract

Rapamycin (Sirolimus) slows aging, extends life span, and prevents age-related diseases, including diabetic complications such as retinopathy. Puzzlingly, rapamycin can induce insulin sensitivity, but may also induce insulin resistance or glucose intolerance without insulin resistance. This mirrors the effect of fasting and very low calorie diets, which improve insulin sensitivity and reverse type 2 diabetes, but also can cause a form of glucose intolerance known as benevolent pseudo-diabetes. There is no indication that starvation (benevolent) pseudo-diabetes is detrimental. By contrast, it is associated with better health and life extension. In transplant patients, a weak association between rapamycin/everolimus use and hyperglycemia is mostly due to a drug interaction with calcineurin inhibitors. When it occurs in cancer patients, the hyperglycemia is mild and reversible. No hyperglycemic effects of rapamycin/everolimus have been detected in healthy people. For antiaging purposes, rapamycin/everolimus can be administrated intermittently (e.g., once a week) in combination with intermittent carbohydrate restriction, physical exercise, and metformin.

Rapamycin (known clinically as Sirolimus or Rapamune) and its analog, RAD001 (everolimus), are FDA-approved drugs widely used in humans. Rapamycin, a natural product, is older, cheaper, better studied, and more available than everolimus, though everolimus is increasingly being used clinically. Both drugs inhibit mTOR complex 1 (mTORC1) by the same mechanism, and their biological effects are identical at equipotent concentrations, taking into account that rapamycin is slightly more potent and has a longer half-life than everolimus^1^. Although the difference in their pharmacokinetics within the human body has been well characterized, that difference has not yet been exploited for clinical purposes, and neither drug is considered superior to the other. As a result, studies with these two drugs complement one another: what has been shown for rapamycin is largely applicable to everolimus and vice versa (at equivalent concentrations). We will use the term rapalogs to encompass both drugs rather than everolimus alone.

## Prevention of insulin resistance and diabetic complications by rapamycin

In diverse studies, rapamycin extends life span in mice (see for numerous refs. ^2–5^). It also improves health not only in rodents, but also in dogs^6^ and primates^7^, and its analog everolimus improves immunity in elderly humans without causing side effects^8,9^.

**Fig. 1:**
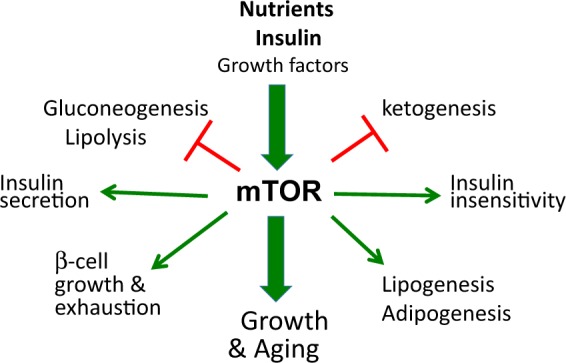
mTOR in glucose and lipid metabolism. Overactivated by nutrients and insulin, mTOR causes noticeable metabolic effects that are reversible by starvation or rapamycin (or everolimus)

Nutrients (glucose and amino and fatty acids), insulin, growth factors, hormones, and oxygen all activate mTOR (target of rapamycin)^10–13^. In turn, mTOR drives both growth and aging^14^ and regulates glucose and lipid metabolism^15–19^. Overactivation of mTOR by nutrients and insulin causes characteristic metabolic alterations (Fig. 1). For example, overactivation of mTOR can cause insulin resistance in humans^20,21^ and has been implicated in type 2 diabetes^11,12,22–25^. Rapamycin prevents insulin resistance caused by nutrient infusion in humans^21^, diminishes insulin resistance in diabetic and hyperinsulinemic rats^26,27^, and normalizes glucose metabolism in diabetic mice^28,29^.

The harmful effects of diabetes are related mostly to its complications, which include nephropathy. In both rats and mice, rapamycin prevents or ameliorates diabetic nephropathy^24,30–41^. For example, hyperactivation of mTOR in podocytes leads to diabetic nephropathy and premature death in mice, preventable by rapamycin^24,30^.

## Prevention and reversal of diabetes by very low calorie diets (VLCDs)

Because mTOR is a nutrient-sensing pathway, it can be deactivated by fasting and severe calorie restriction (CR), which exert metabolic effect that are somewhat similar, but not identical, to those of rapamycin^42^. Fasting and VLCDs are used to treat type 2 diabetes^43–50^. VLCDs prevent and reverses diabetes, especially at its early stages. Likewise, rapid and sustained weight loss can resolve or reverse type 2 diabetes in most patients^43–51^. VLCD-induced remission of diabetes depends in part on disease duration, probably because the loss of beta cells in cases of long-duration diabetes renders the disease less reversible. In one study, for example, 87% of patients with short-duration, but only 50% of those with long-duration, diabetes achieved nondiabetic fasting plasma glucose levels by week 8 of a VLCD^44^. In the latest study, VLCD therapy achieved persistent nondiabetic blood glucose control in 46% of patients with up to a 6-year history of diabetes^51^. VLCD treatment is effective and simple. Using a VLCD, motivated patients are able to reverse type 2 diabetes at home^43^. Notably, rapamycin more potently inhibits mTOR than does fasting, especially in old age^52–54^.

## Puzzling contradictions

In 2012, a Science paper entitled “Rapamycin-induced insulin resistance is mediated by mTORC2 loss and uncoupled from longevity^55^” turned everything upside down. Data were misinterpreted to indicate rapamycin causes diabetes. Because the paper was published in a high profile journal, basic researchers believe that rapamycin is harmful and causes diabetes, which prompted calls for development of rapamycin-like drugs without rapamycin effects. In fact, however, this paper does not show that rapamycin causes type 2 diabetes; it shows that prolonged treatment with rapamycin causes glucose intolerance and insulin resistance in mice^55^, which is in agreement with earlier studies^56–58^. Furthermore, the study showed that these metabolic alterations were associated with increased longevity, indicating better health^55^.

In humans, diagnosis of diabetes depends on the arbitrary choice of a threshold for fasting blood glucose: it was 140 mg/dl before 1997 and 126 mg/dl after 1997. But what is the arbitrary diagnostic threshold in mice? It is not defined. Is the slight increase in fasting glucose shown in Fig. 1g of Lamming et al.^55^ sufficient for a “diagnosis” of diabetes in mice? Does such hyperglycemia decrease life span or cause nephropathy? It does not^55^. As we will discuss next, similar glucose intolerance and insulin resistance can also be caused by prolonged fasting and extreme VLCD. Prolonged fasting and starvation cause a condition well known in the past but unknown to modern researchers: starvation pseudo-diabetes.

## Starvation pseudo-diabetes

In 1846, Claude Bernard detected glycosuria (glucose in urine) in a rabbit fed carrots after several days of fasting (cited in ref. ^59^). Later, Claude Bernard wrote: “If a man or an animal is fasted for some time, and then given a good meal with an abundance of carbohydrate, glucose will appear in the urine^59^.” This condition has been referred to as starvation diabetes, hunger diabetes, or pseudo-diabetes^59,60^.

**Fig. 2:**
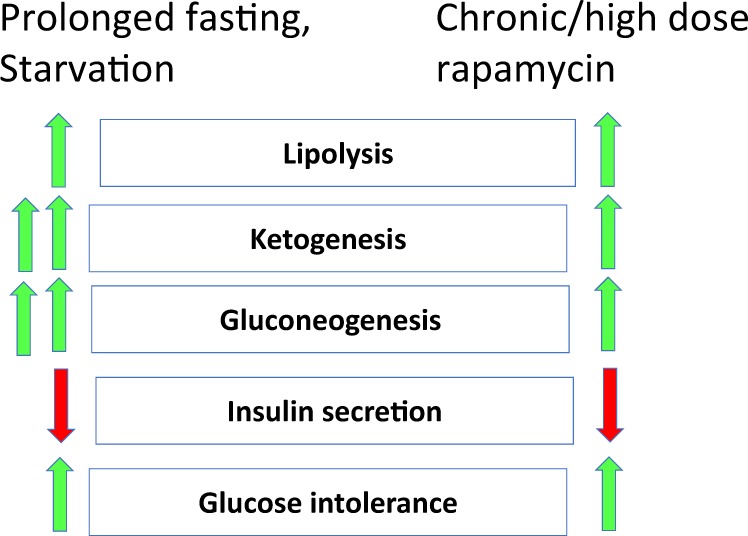
Comparison of starvation- and rapamycin-induced benevolent pseudo-diabetes. Common alterations caused by prolonged fasting/starvation or chronic treatment with rapamycin (or everolimus). Green arrow—upregulation; red arrow—downregulation

**Fig. 3:**
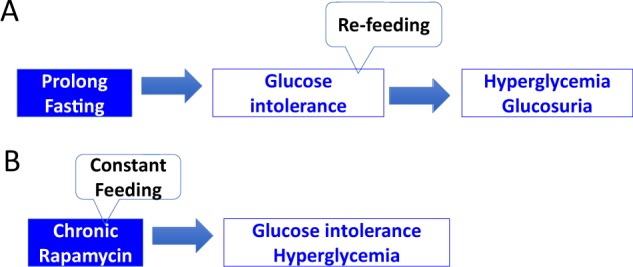
From glucose intolerance to hyperglycemia. **a** Starvation causes glucose intolerance, but levels of blood glucose are low due to glucose deprivation. Resuming carbohydrate feeding causes hyperglycemia associated with glycosuria, which has been termed “starvation pseudo-diabetes.” **b** Chronic rapamycin treatment is accompanied by constant feeding, so glucose intolerance is associated with mild hyperglycemia, reversible by rapamycin discontinuation

During starvation or prolonged fasting, glucose utilization by nonbrain tissues is inhibited in order to feed the brain. Prolonged fasting is characterized by low insulin levels, gluconeogenesis, lipolysis, ketogenesis and ketosis (ketone bodies in the blood), glucose intolerance, and hepatic resistance to insulin (Fig. 2a). When a starved animal is fed with glucose, it cannot utilize the glucose (glucose intolerance), leading to transient glycosuria (glucose in the urine) and polyuria (high urine volume) (Fig. 3a).

## Rapamycin-induced hyperglycemia

Given that rapamycin is a starvation- or CR-mimetic^42,61,62^, its metabolic effects can be viewed as “starvation-mimicking side effects^63^." In a series of publications, I advanced the hypothesis that in animals (and humans) rapamycin can cause a reversible and benevolent condition, identical to starvation pseudo-diabetes^64–66^ (Figs. 2b and 3b). If so, this condition may, in theory, prevent development of genuine type 2 diabetes and its complications. For example, rapamycin prevents diabetic nephropathy^24,30–41^. The results of recent studies are consistent with the idea that rapamycin-induced metabolic alterations are reversible and beneficial in nature^3,28,67–71^. Hyperglycemia may be a marker of beneficial processes, given that rapamycin ameliorates nephropathy, despite elevating blood glucose levels in a mouse model of type 2 diabetes^39^.

The notion of benevolent insulin resistance also resolves the insulin resistance paradox; that is, insulin resistance is associated with both decreased or increased life span. Insulin resistance due to activation of mTOR shortens life span (see Fig. 1 in ref. ^65^), whereas insulin resistance due to inhibition of mTOR increases life span (see Fig. 3 in ref. ^65^). Simply stated, “Insulin resistance associated with TOR overactivation is bad, … but … insulin resistance associated with inactive TOR is good^65^." This is the mTOR-centric view on glucose metabolism^66^. Detrimental metabolic alterations should have detrimental consequences, such as diabetic complications, but there is no evidence that rapamycin-induced glucose intolerance is detrimental. On the contrary, rapamycin improves nephropathy in diabetic mice, despite increasing blood glucose levels^39^.

Rapamycin- and everolimus-induced hyperglycemia is reversible by drug discontinuation or even by metabolic adaptation^3,69^. Is such reversible hyperglycemia indicative of type 2 diabetes or pseudo-diabetes, or is it simply a reversible unintended effect? In transplant patients, who often develop diabetes, even without rapamycin/everolimus^72^, any hyperglycemia above 126 mg/dl is called diabetes. In cancer patients, by contrast, hyperglycemia is considered to be a side effect, not diabetes. Numerous drugs, including rapamycin (Sirolimus), everolimus, and temsirolimus (a rapamycin prodrug), can cause this side effect. In these cases, the severity of the hyperglycemia, like any other side effect, is assigned a grade 1–4. In most cases, rapamycin/everolimus-associated hyperglycemia is grade 1–2 and does not lead to treatment interruption^73,74^.

## Comparing starvation- and rapamycin-induced glucose intolerance

Prolonged fasting and chronic treatment with rapamycin may cause similar metabolic alterations, including dyslipidemia (Box 1). Dyslipidemia is mediated in part by lipolysis, which increases levels of circulating free fatty acids, providing a substrate for ketogenesis and causing insulin resistance^75,76^. Coupled with inhibition of insulin secretion, insulin resistance promotes gluconeogenesis and ketogenesis. Dyslipidemia is the most common side effect of rapalogs. Similarly, prolonged fasting increases blood lipid levels in healthy subjects^77–81^.

Prolonged fasting causes glucose intolerance, which translates to hyperglycemia when glucose is consumed. During glucose deprivation, glucose levels are low, of course. Reintroducing glucose to the diet leads to excessively high blood glucose levels and glycosuria (Fig. 3a). [Note: By definition glucose intolerance implies hyperglycemia after glucose consumption]. During treatment with rapamycin, animals are constantly fed, so glucose intolerance is accompanied by hyperglycemia, which reversed by rapamycin discontinuation (Fig. 3b). It is predictable that rapamycin treatment during glucose deprivation, such as occurs during prolonged fasting or starvation, or a VLCD, or ketogenic diet (KD), will not elevate glucose levels. Instead, glucose levels will be as low as during starvation. The therapeutic potential of combining rapalog treatment with CR awaits investigation.

Box 1 Can we link rapamycin and everolimus to diabetes in humans?

- In transplant patients: careful analysis has revealed no solid evidence ^91,83^ that rapamycin or everolimus alone (independently of cyclosporine/tacrolimus coadministration) increases the risk of diabetes
- In cancer patients: hyperglycemia is not considered to be diabetes, but a reversible side effect
- In healthy humans, including the elderly, hyperglycemia has not been noticed so far

## Rapamycin in renal-transplant patients

When basic scientists claim that rapamycin causes diabetes in humans, they cite a paper by Johnston et al., which is a retrospective analysis of the association between Medicare billing for diabetes with rapamycin use in renal-transplant patients^82^. What this study actually demonstrates is that rapamycin-containing drug combinations are associated with more Medicare billing for diabetes than are combinations without rapamycin^82,83^. Detailed analysis suggests that rapamycin treatment is associated with diabetes when combined with a calcineurin inhibitor, which is a well-known diabetogenic drug^82,83^. Rapamycin and calcineurin inhibitors interact and increase each other's concentrations.

Other studies do not reveal an association between rapamycin and diabetes, and even show that use of rapamycin instead of other drugs decreases the incidence of diabetes^84–91^. For example, a shift from calcineurin inhibitors to rapamycin improves metabolic parameters and insulin requirements in patients with new-onset diabetes after transplantation, which can be resolved in 80% of patients^91^. In some studies, rapamycin has shown a tendency to prevent diabetes after kidney transplantation^86,88^. In addition, a large clinical trial did not find that rapamycin increases the incidence of diabetes^87^, and in a pilot study, rapamycin did not cause diabetes in kidney transplant recipients during 3 years of follow up^89^. Moreover, transplant patients with pre-existing diabetes were successfully treated with rapamycin without worsening their diabetes^90^.

Outstanding analyses by Veroux et al.^91^ and Pavlakis et al.^83^ reconciled conflicting results from Johnston et al. and Kasiske et al., who used the same database^85,82^. Pavlakis et al. suggested that, by itself, rapamycin is not associated with diabetes, though its combination with tacrolimus makes the known diabetogenic effect of tacrolimus worse^83^. Needless to say, tacrolimus will not be used for antiaging purposes because it has nothing to do with aging.

Finally, obesity is a major risk factor for type 2 diabetes in transplant patients, just as it is for the general population. Rapamycin treatment decreases weight and adiposity in transplant patients^92^. There is thus still no evidence that, by themselves, rapamycin and everolimus are diabetogenic in transplant patients.

## Rapamycin/everolimus in cancer patients and generally healthy individuals

In cancer patients, rapamycin, everolimus, and temsirolimus can induce reversible and mild (grade 1–2) hyperglycemia that usually does not require drug discontinuation^73,74^. Although observed in a minority of patients, this hyperglycemia is associated with an enhanced anticancer response to everolimus^74^. According to a meta-analysis, the risk of hyperglycemia associated with everolimus treatment varies from 3% in breast cancer to 27% in renal cell carcinoma^73^. Rapamycin/everolimus are administered to cancer patients at very high doses. In one study, 60 mg of rapamycin given once a week was associated with diarrhea, though patients were able to continue therapy^93^. It is noteworthy that these doses are ten times higher than the weekly doses suggested for antiaging therapy by Dr Alan Green (https://joshmitteldorf.scienceblog.com/2016/06/13/rapamycin-redux/). In placebo-controlled studies, the side effects of rapalogs were manageable with dose reduction and interruption^94^.

Patients with systemic lupus erythematosus treated daily with 2 mg of rapamycin for 12 months^95^ developed mild anemia, leucopenia, and hyperlipidemia, but hyperglycemia was not reported^95^.

In generally healthy elderly, treatment with 1 mg/daily of rapamycin for 8 weeks^96^ as well as treatment with everolimus (RAD001) plus pan-mTOR inhibitor daily for 6 weeks^9^ was safe and without reported hyperglycemia^8,9,96^. High single doses did not cause hyperglycemia in healthy volunteers^97^. And there was no change in glucose levels after rapamycin overdose^98^.

## Rediscovery of starvation pseudo-diabetes in humans

CR improves health in humans^48,99^. Nevertheless, a remarkable study by Fontana et al. reported that 40% of individuals practicing severe CR exhibited “diabetic-like” glucose intolerance^99^ and speculated that a similar sort of “insulin resistance” slows aging in mice^99^. In one study, the effect of a 60-h fast on insulin sensitivity depended on the prestarvation status: the insulin sensitivity of insulin-resistant subjects stayed the same or increased slightly, whereas more insulin-sensitive subjects tended to show a decrease in insulin sensitivity^100^. Startlingly, a study entitled “Starvation diet and very low calorie diets may induce insulin resistance and overt diabetes mellitus” described seven obese individuals, who developed diabetes during a strenuous weight reduction program^101^. How can weight loss and a VLCD cause diabetes? A starvation diet or VLCD is the most powerful treatment option to prevent, treat, and reverse type 2 diabetes. Curiously, these authors also claimed that this phenomenon “has not previously been reported in the medical literature^101^.” Actually, it was first reported in 1848 by Claude Bernard, who observed it in a fasting rabbit and coined the term starvation pseudo-diabetes^59^. It was subsequently noticed again by Lehmann in fasting dogs in 1874^60^. “Since then the phenomenon of starvation diabetes has been repeatedly reported in one connection or another^60^.” In 2011, it was proposed that mTOR inhibitors such as rapamycin may induce pseudo-diabetes instead of type 2 diabetes^64^. KDs also induces pseudo-diabetes and are associated with better health and reversal or prevention of genuine type 2 diabetes.

## Ketogenic diet

The beneficial nature of pseudo-diabetes brings up startling questions. Should we intentionally cause pseudo-diabetes to improve health and can pseudo-diabetes be reliably induced? Yes, pseudo-diabetes can be induced by a KD. Ketosis is a prominent feature of diabetes, and, by definition, a KD induces ketosis. In addition, a KD may cause other markers of diabetes in some rodent models. In rats and mice, for example, a KD causes insulin resistance and glucose intolerance^102–106^. It also increases levels of atherogenic lipoproteins and decreases levels of the antiatherogenic HDL cholesterol^107^. In long term, a KD may increase levels of cholesterol, triglycerides (dyslipidemia), and inflammation markers, associated with glucose intolerance and insufficient insulin secretion^105^. In rodents fed a KD, a carbohydrate meal induces hyperglycemia, but the pseudo-diabetes is rapidly reversed upon cessation of the KD^102^. Is KD-induced pseudo-diabetes detrimental or beneficial? KDs are being increasingly employed to treat obesity, cancer, neurological diseases^108–110^, and type 2 diabetes^111–113^. KDs extend both the life span and health span of mice^114^. [Note: More on KDs and pseudo-diabetes in a forthcoming article entitled “The mystery of the ketogenic diet: benevolent pseudo-diabetes.” Cell Cycle, 2019, in press].

## Rapamycin in diabetic rodents

Can rapamycin be used in cases of overt diabetes? On the one hand, rapamycin can decrease beta cell function and thus decompensate diabetes. On the other hand, rapamycin can decrease beta cell hyperfunction, preventing cell failure in the long run. In five murine models of diabetes, rapamycin did not exacerbate diabetic phenotypes^28^. Rapamycin increased insulin sensitivity and reduced weight in three models, decreased hyperinsulinemia in two models, and elevated hyperglycemia in one model^28^. In other studies, rapamycin decreased body weight and improved insulin sensitivity^115,116^. Intermittent rapamycin administration can improve insulin sensitivity and decrease insulin levels in mice on a high-fat diet^70,71^. In severely diabetic fat sand rats (*P. obesus)*, which exhibit extreme hyperinsulinemia, compensation depends solely on insulin overproduction; consequently, rapamycin may cause decompensation. Thus, by abolishing hyperinsulinemia, rapamycin prevents beta cell adaptation to hyperglycemia^117^. However, the fat sand rat model is so peculiar that it has little relevance to human type 2 diabetes, especially given that decompensated diabetic humans would probably be treated with exogenous insulin.

In all mouse strains but one, rapamycin increases the life span. The exception is the mutant short-lived mice (C57BL/KsJ-*lepr*^*db/db*^), which exhibit severe diabetes and other abnormalities. Rapamycin shortens the life span of these mice^118^, though it protects them from both diabetic nephropathy^32^ and cancer^118^. [Note: In parental “normal” mice of this strain, rapamycin prolongs life^119^]. But should rapamycin prevent death from all causes? Of course not. Although rapamycin can delay death caused by age-related diseases, not all diseases are aging related. Rapamycin would be useless and even harmful in cases of trauma, appendicitis (suppurative inflammation of appendix), and malnutrition. In such cases, rapamycin may, in theory, increase mortality, especially in young subjects. Consistent with that idea, rapamycin further shortens the life of C57BL/KsJ-*lepr*^*db/db*^ short-lived mice, which die early from suppurative inflammation (pus in multiple organs). In humans, examples of suppurative inflammation are appendicitis and pancreatitis, which are not aging-related diseases. Type 2 diabetes does not cause appendicitis, and suppurative inflammation of all organs is hardly ever seen in humans. Why would rapamycin extend life span in mice that die young from the pus in the organs?

In summary, the effects of rapamycin in peculiar short-lived mice that die from suppurative inflammation cannot be extrapolated to humans. Rapamycin and everolimus are safely used by millions of patients, and no results from diabetic short-lived mice change that simple fact. Clinical data are accumulating, and although some correlations between rapamycin/everolimus and diabetes have been reported, there is no evidence that these drugs per se cause genuine type 2 diabetes. Even if we find additional peculiar genetically manipulated rodent models that exhibit paradoxical effects of rapamycin, this will not affect its clinical application. A large body of data from humans are already available and show that both rapamycin and everolimus are safe. And after all, rapamycin prevents and treats diabetic nephropathy in rats and mice^24,30–41^. Analogously, in one study, dietary restriction shortened the life spans of multiple strains of inbred mice^120^. Yet dietary restrictions and VLCDs are powerful modalities for preventing and reversing diabetes and improving overall health.

## Function, hyperfunction, and loss of beta cell function

**Fig. 4:**
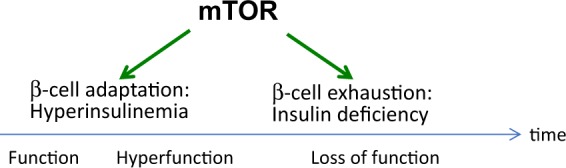
Biphasic effects of mTOR on beta cells. mTOR initially stimulates beta cell growth and function, thereby enhancing insulin production. Chronic activation of mTOR leads to beta cell hyperfunction and eventually to their exhaustion (loss of function)

mTORC1 stimulates beta cell growth, causing hyperplasia, hypertrophy, and increased beta cell function—namely, insulin production^121,122^. Chronically increased beta cell function (hyperfunction) is initially adaptive, but eventually may lead to the cells’ exhaustion and loss of function^122–124^. Consequently, mTORC1 may exert a biphasic effect on beta cell function^64,124^ (Fig. 4). This scenario is in agreement with the hyperfunction theory^125,126^, which posits that aging and age-related diseases are associated with cellular (e.g., hypertrophy, hyper-secretory phenotypes, or SASP) and systemic (e.g., hypertension, hyperlipidemia, pro-inflammation, and hypercoagulation) hyperfunction. mTOR-driven hyperfunction eventually leads to organ damage, but it can be suppressed by rapamycin/everolimus^125^. This suggests that, in the case of diabetes, although rapamycin may decrease beta cell function in a short run, it preserves beta cell function in the long run (Fig. 4). Similarly, mTOR hyperactivity (hyperfunction) in renal podocytes leads to nephropathy^24,30^, which is preventable by rapamycin, despite the hyperglycemia^24,30,39^. By deactivating mTOR in the retina, kidneys and other organs, rapamycin/everolimus render hyperglycemia benevolent and prevent diabetic complications^65,66^.

## Benevolent insulin resistance with low mTOR activity

Inhibition of mTORC1 increases insulin sensitivity^11,12,21–27,127^, while inhibition of mTORC2 causes insulin resistance in mice^55^. In fact, a single dose of pan-mTOR inhibitor, which directly inhibits mTORC2, induces insulin resistance in mice, whereas the mTORC1 inhibitor rapamycin has no effect^128^. That said, although mTORC2 is not a target of rapamycin, it is deactivated indirectly by prolonged rapamycin treatment^129^. In a human study, however, a pan-mTOR inhibitor did not cause hyperglycemia^9^. Furthermore, a mechanistic study in humans showed that prolonged treatment with rapamycin causes hyperglycemia by decreasing insulin production^130^, and insulin production depends on mTORC1. Thus, inhibition of mTORC2 by rapamycin has not yet been proved in humans.

Lamming et al. concluded that rapamycin prolongs life span, despite inducing diabetes-like symptoms^55^. As I previously discussed, perhaps life span is extended “owing to,” not “despite,” these symptoms^65^. Once again, whereas type 2 diabetes shortens life span, rapamycin prolongs it (despite, or maybe owing to, benevolent metabolic alterations). Type 2 diabetes is associated with activated mTOR and leads to diabetic complications, which are the worst aspect of the disease. By contrast, rapamycin prevents these complications (see for refs. ^65,66^). In fact, chronic treatment of C57BL/6 mice with rapamycin causes a diabetes-like syndrome without complications and is associated with life span extension and improved health^55^.

## Why rapamycin-induced metabolic alterations vary

In many studies, chronic daily administration and, especially, intermittent or low-dose administration of rapamycin or everolimus did not cause hyperglycemia^7,54,70,131–134^. In some studies, chronic treatment with rapamycin caused insulin resistance or glucose intolerance without insulin resistance, but also insulin sensitization with glucose intolerance^28,39,70,122,135^. The effects of rapamycin depend on its dose, duration of administration (especially), route of administration, time of administration, species and/or strain, sex, diet, obesity, and other factors. And beta cell function often determines the outcome. In mice, insulin resistance can be explained by prolonged deactivation of mTORC2 during treatment^55,129^. Insulin resistance (due to mTORC2 deactivation) in addition to decreased insulin secretion (due to mTORC1 deactivation) may contribute to glucose intolerance.

VLCDs prevent and reverse type 2 diabetes and obesity^43–50^. Achieving remission depends in part on the capacity of beta cells for recovery^51^. Similarly, the ability of rapamycin to prevent diabetes may be counteracted by its effects on beta cells under conditions of high insulin demand^136,137^. In brief, the antidiabetogenic effects of rapamycin may depend in part on beta cell status^28^. To further assess the beneficial nature of pseudo-diabetes in rodents, diabetes should be induced by rapamycin or a diabetogenic diet. A testable prediction is that diabetic pathology will be observed only in the later group, which will exhibit shorter life spans than the rapamycin group.

## Intermittent rapamycin or a single high dose

Intermittent rapamycin administration (pulse treatment) was proposed in 2008, as a means of rejuvenating stem and wound-healing cells, thereby improving wound-healing instead of impairing it, as chronic treatment with rapamycin did^138^. Intermittent administration (e.g., once a week) can be considered a single dose repeated over time. In mice, a single dose of rapamycin does not cause glucose intolerance, but a single dose of a dual mTORC1/mTORC2 antagonist does causes it^128^. Consistent with that finding, weekly treatments with rapamycin for 22 weeks inhibited mTORC1 and protected against insulin resistance in C57BL/6 mice fed a high-fat diet, whereas mTORC2 activity remained intact^70^. Higher single doses of rapamycin can be used when administration is intermittent than when it is chronic, and it appears that it is the peak concentration of rapamycin that is especially beneficial^2^. This is in part because high peak levels enable rapamycin to cross the blood–brain barrier. For example, rapamycin prevents obesity associated with hyperactive mTOR in hypothalamic POMC neurons^139^. Furthermore, intracerebroventricular injection of rapamycin is sufficient to decrease weight gain^140^. Intraperitoneal injections of rapamycin produce high blood levels of the drug and prevent weight gain when administered every other week. Indeed, a single intraperitoneal administration decreases weight gain for 10 weeks without additional injections^140^. By contrast, administration of rapamycin by oral gavage did not prevent weight gain^132^.

## Conclusion and further directions

Despite the fact that rapamycin is a FDA-approved drug taken by millions of patients, some basic scientists believe that rapamycin causes deleterious metabolic alterations or even diabetes and, therefore, cannot be safely used in humans as an antiaging drug. Actually, rapamycin prolongs life and improves health overall. It prevents diabetic complications in rodents. A diabetes-like condition that improves health and prolongs life may not be a disease, but rather a pseudo-disease or even a disease preventer. Similarly, prolonged fasting, VLCDs, severe CR, and KDs can cause benevolent pseudo-diabetes, which may counteract type 2 diabetes. Likewise, rapamycin may induce pseudo-diabetes that is reversed upon the drug’s discontinuation^28,67-70^.

The notion of *starvation* pseudo-diabetes is applicable to prolonged fasting, extreme VLCDs, and KDs, as well as to rapamycin, everolimus, and other rapalogs. Without the concept of starvation pseudo-diabetes, some reported observations, such as induction of diabetes during strenuous weight reduction programs, would be unexplainable^101^. How can weight loss cause diabetes? After all, a starvation diet or VLCD is the most effective way to prevent and reverse type 2 diabetes.

Up to now, there have been no reports of rapamycin/everolimus causing hyperglycemia in generally healthy elderly individuals taking these drugs daily/chronically^8,9,96^. Treatment with very high doses of rapamycin/everolimus/temsirolimus causes hyperglycemia in some cancer patients^73^, but this reversible dose-dependent effect is not viewed as diabetes, but as a side effect^73,74^. In renal-transplant patients, an association between rapamycin and diabetes was revealed in a study analyzing a large group (20,124 patients) in order to achieve a statistical power^82^. This retrospective study was based on Medicare billing for antidiabetic drugs related to rapamycin use^82^. However, this association was primarily due to an interaction between rapamycin and tacrolimus (a diabetogenic drug) ^83,91^. Other studies have not found an association between rapamycin and diabetes^84–91^. Notably, the incidence of diabetes among transplant patients is up to 50–70%, even without rapamycin treatment. Moreover, the incidence of “transplant-associated hyperglycemia,” encompassing all types of abnormal glucose homeostasis, is up to 70% 1 year after transplantation ^72^. In those taking rapamycin, this may be benevolent pseudo-diabetes rather than type 2 diabetes. That would explain the puzzling observations that conversion to rapamycin resolved diabetes in 80% patients ^91^, and that conversion from calcineurin inhibitors to mTOR inhibitors stabilizes diabetic and hypertensive nephropathy after liver transplantation^141^.

As an antiaging treatment, rapamycin or everolimus could be administered intermittently, which would not be expected to cause hyperglycemia. I suggest that intermittent rapamycin administration be combined with simultaneous intermittent fasting or VLCD, as well as physical exercise. If potential hyperglycemia is still a concern, metformin can be used to counteract it^142^. In humans, a combination of rapamycin and metformin was well tolerated without unexpected side effects^143^. Rapamycin/everolimus with metformin may have numerous benefits, including anticancer and antiaging effects^144^.
